# Screen viewing behavior and sleep duration among children aged 2 and below

**DOI:** 10.1186/s12889-018-6385-6

**Published:** 2019-01-14

**Authors:** Bozhi Chen, Rob M. van Dam, Chuen Seng Tan, Hwee Ling Chua, Pey Gein Wong, Jonathan Y. Bernard, Falk Müller-Riemenschneider

**Affiliations:** 1 Saw Swee Hock School of Public Health, National University of Singapore and National University Health System, Singapore, Singapore; 2 Harvard T.H. Chan School of Public Health, Harvard University, Boston, MA USA; 3 Department of Family Medicine, Yishun Polyclinic, Singapore, Singapore; 4 Jurong Polyclinic, National Healthcare Group Polyclinics, Singapore, Singapore; 5 Singapore Institute for Clinical Sciences, Agency for Science, Technology and Research (A*STAR), Singapore, Singapore; 6 Institute for Social Medicine, Epidemiology and Health Economics, Charite Univeristy Medical Centre Berlin , Berlin, Germany

**Keywords:** Screen viewing, Sleep, Television, Mobile device, Sedentary, Young children

## Abstract

**Background:**

Few studies have investigated the association between screen viewing (SV) and sleep duration among young children. This study aims to examine the association between total and device-specific SV and sleep duration among children aged 2 and below.

**Methods:**

We conducted a cross-sectional study of 714 Singaporean children aged 2 years and below. Parents were recruited during routine well-child clinic visits from two national polyclinics. In Singapore, all parents visit well-child clinics with their children at regular intervals for routine check-ups and vaccinations. Socio-demographic characteristics, duration of total and device-specific SV, and sleep duration were reported by parents via interviewer-administered questionnaires. Multiple linear regression analysis was used to assess associations between various types of SV and sleep duration, adjusted for socio-demographic variables. Due to significant interaction between SV and age, stratified analyses for children aged less than 6 months and those aged 7–24 months were performed.

**Results:**

The prevalence of daily SV among children was 53.1%; 28.3% in children up to 6 months and 73.8% in children aged 7 to 24 months. TV viewing was reported for 44.3% of all children and mobile device SV for 30.1%. Children’s average sleep duration was 13.9 (SD = 3.5) hours daily and younger children had longer sleep duration than older ones (up to 6 months: mean = 15.6 h, SD = 3.9; 7–24 months: mean = 12.4 h, SD = 2.2; *P* < 0.01). In the regression analysis among all children, each 1 h per day increment in total SV was significantly associated with 0.26 h shorter sleep duration with similar significant associations for TV (β = − 0.28 h, 95%CI: -0.50, − 0.06) and mobile devices (β = − 0.35 h, 95%CI: -0.61, − 0.09). Stratified analysis revealed significantly greater reductions in sleep with higher SV among children aged 6 months and below (β = − 0.73 h, 95%CI: -1.12, − 0.34), while associations were weaker in older children (β = − 0.13 h, 95% CI: -0.24, − 0.01).

**Conclusions:**

This study provides evidence for a substantial association between longer SV and shorter sleep duration among very young children. These associations appeared stronger among children aged 6 months and below as compared with those aged 7 to 24 months. Further studies are warranted to confirm our findings.

**Electronic supplementary material:**

The online version of this article (10.1186/s12889-018-6385-6) contains supplementary material, which is available to authorized users.

## Background

Screen media usage has become increasingly prevalent in daily life with children beginning to use screen media at early age. In Japan, by 18 months old, 86% of children spend over 1 h watching television (TV) a day [1]. In Australia, children under 4 years of age spend more than 2 h watching TV per day on average [2]. Similarly, in the United States (US), 90% of children at age 2 regularly watch TV or videos, with the average duration exceeding 1.5 h a day [3, 4].

Given the high prevalence of screen viewing (SV) among children worldwide, the impact of SV on children’s health has been of rising concern. However, the current information on associations between SV and health outcomes among children is mixed. Studies have shown that media exposure is more likely to have detrimental impacts on children’s health and development before the age of about 2.5 years than after [5, 6]. Potential adverse effects span several developmental domains including delayed cognitive and language development [7, 8], impaired social interaction [9, 10], attention deficits [8, 11, 12], and behavioral problems such as violent behavior and aggression [13–15]. In addition to developmental delay, higher SV exposure has been associated with negative impacts on vision [16–18] and a greater likelihood of being overweight or obese in late childhood and adolescence [19, 20]. On the other hand, results from some studies have suggested benefits of screen media usage among children. For example, children may benefit from virtual visits to their relatives through live video chat [21] and watching educational media with parents might contribute to general language development [22]. The American Academy of Paediatrics (AAP) has recently modified its SV guidelines. Instead of recommending no SV for children before age 2, it now emphasizes the importance of high-quality programming and parents’ accompanying [23] their children while SV. AAP recommends no SV of children before 18 months except for video-chatting, for instance to prompt interactions with distant family members and parental supervision and support are necessary for children to understand what they are seeing [23]. AAP has reported no evidence that infants can actually benefit from media the way they do from live social interactions.

Among adults, inadequate sleep has been independently associated with overweight and obesity and the development of type 2 diabetes as it may lead to higher energy intake, less physical activity, increased cortisol levels and reduced insulin sensitivity [24–27]. The importance of sleep in memory-related domains (including verbal, emotional and procedural development) has been exhibited in both human and animal studies [28]. Children may be more vulnerable to negative effects of inadequate sleep as they gain complexity in domains as cognition and language skills during their neuro-development. There is increasing evidence that suggests inadequate sleep quantity in early life is associated with negative consequences at a later age including cognitive and behavioral deficits which, in turn, affect daytime functioning [29]. Other consequences relate to detrimental health outcomes such as diabetes [30], cardiovascular disease [31], obesity [32] and depression [33]. The current understanding of mechanisms of sleep in young children is bolstered by recent research suggesting that sleep is crucial for the brain to remember and learn by resetting the steady built-up synaptic connectivity during wake time [34].

Screen media usage may indirectly influence sleep because brightly lit screens may lead to hyperarousal resulting in automatic activation increase [35]. Screen media usage may directly limit sleep duration by delaying or interrupting sleep time [36]. There is evidence supporting the hypotheses that increased screen time is associated with shorter sleep duration [37–40], night awakenings and disturbed sleep [41–45]. Most studies investigating associations of SV behavior with inadequate sleep have focused on adults, adolescents or school-age children, while research on very young children is limited. A recent longitudinal study from the US reported that greater TV viewing during infancy was associated with shorter sleep duration [46] in mid-childhood. The study provided new evidence for the detrimental effects of TV viewing on sleep but did not explore the contribution of other forms of screen media (e.g. mobile phones) to total SV or their impact on sleep duration among very young children. Considering the potentially harmful effects of SV behavior, the scarcity of evidence related to the changing patterns of SV and how other types of screen media, including computers, video consoles and mobile devices may affect sleep and other health related outcomes in very young children is an important limitation of the existing scientific literature.

With a predominantly Asian population in one of the most wired countries in the world [47], this study in Singapore aims to address these gaps by examining the association of total and device-specific SV and sleep duration in children aged 2 years and below.

## Methods

### Study population

We conducted a cross-sectional study by recruiting parents of children aged up to 2 years in February 2014. The study has previously been described in detail [48]. Briefly, parents were recruited when they brought their children to government polyclinics for routine developmental assessments and mandatory vaccinations. They were recruited from two National Healthcare Group polyclinics, out of total 18 polyclinics in Singapore over a 7-day period. As Singapore’s National Childhood Immunization Program for children up to 2 years has a take-up rate of more than 97%, children attending this program are good representation of the children population in the region at a certain point of time [49]. Parent-child dyads were invited to participate in this study if they met the following inclusion criteria: 1) children were 2 years and below; 2) children were healthy and brought for well-child health visits to the two National Healthcare Group Polyclinics. Non-Singaporeans were excluded.

### Ethics statement

On recruitment, verbal informed consents were obtained from all participating parents and questionnaires were administered using participants’ preferred language (English, Chinese, Malay or Tamil) by trained interviewers during clinic visits. The study was approved by the Singapore National Health Care Group Domain Specific Review Board (DSRB).

### Measurements

The questionnaire was developed based on a comprehensive review of the literature on determinants and consequences of SV. Parents were asked to report the amount of time their children spent actively watching TV/DVD, computers, video games consoles and mobile devices (including mobile phones, tablets and handheld video game devices) at home on weekdays and weekends (see Additional file 1 for the items). This question emphasized foreground viewing, in which children are actively watching screen devices, to generate a conservative estimate of children’s screen media exposures. It is also believed that parents will report their children’s foreground exposure more accurately as compared with background viewing (for example, a TV is turned on but the children are not actively viewing it). Children’s foreground screen time recalled by parents has been shown to be accurate and reproducible [50]. Although parent reporting has more errors than objective measurements (i.e. video-recorder), it has been shown to exhibit no systematic bias [51]. The daily device-specific SV time was calculated as follows: ((weekdays × 5) + (weekends × 2))/7. Total SV was then determined by adding up the daily SV time for each type of SV device. We also classified total and device specific SV into 4 groups: 0 h, < 1 h, 1–2 h, ≥2 h.

Daily total sleep duration, including daytime and nighttime sleep, was assessed by one question: “How much time in a day does your child spend on sleeping?”. This question was derived from the Children’s Sleep Habit Questionnaire (CSHQ), a commonly used paediatric sleep screening tool, which has been validated among infants and children and used widely in other studies [52–54]. Sleep assessed by CSHQ has shown to have a moderate correlation with that observed by the Infant Sleep Chronogram [54]. Parents’ education, marital status, ethnicity and housing type were recorded together with the child’s age and sex.

Cases beyond the lower inner fence, which equal 25th Percentile - (1.5 * Interquartile Range) were defined as outliers [55]. Thus, the participants who reported a sleep duration of less than 6 h per day were excluded from analysis.

### Statistical analyses

Socio-demographic and baseline characteristics were expressed as means and standard deviations (SD) for continuous variables, and frequencies and percentages for categorical variables. Fisher’s exact test (for categorical variables) and Student’s t-test (for continuous variables) were performed to compare differences between two age groups (“up to 6 months” and “7–24 months”). The associations of categorical SV (total and device specific SV) with sleep duration were tested using linear regression models, with children having no corresponding SV as the reference groups. Linear effect of continuous SV (total and device-specific SV) on sleep duration was also examined. In multiple linear regression analysis, we further adjusted for potential confounders including child’s age, sex and race, caregiver, marital status of parents, housing type and paternal and maternal educational levels.

To test for possible interaction between SV and age, sex and ethnicity, an interaction term was added to the multivariable regression model one at a time. Only age was found to have significant interaction with SV. Therefore, stratified analyses by age group were conducted. All tests conducted were two-sided and considered significant if *P* < 0.05. All statistical analyses were performed using Stata, version 14.0 (StataCorp, 2015).

## Results

Among the 1061 parent-child dyads approached during their visit to the polyclinics, 794 met the inclusion criteria and 722 (91.0%) provided verbal consent and completed the survey giving us data for 722 children. Eight parents who reported a sleep duration of less than 6 h per day were excluded from analysis. The current analysis includes 714 children.

### Characteristics of the study population

Child and parental characteristics of the study population are presented in Table 1. Among all children, 55.0% were male and 58.7% were of Chinese descent, with the remainder being mostly of Indian or Malay descent. The majority of children (55.0%) were between 7 and 24 months. Nearly all parents were married (98.0%) and 73.4% participants reported that they took care of their children by themselves. 79.4% of fathers and 75.8% of mothers completed post-secondary education and above, and the majority of the study population (95.7%) lived in public government housing, where most Singaporeans reside [56]. In general, socio-demographic characteristics were well balanced between the two age groups.

**Table 1:** Characteristics of participants included in the overall analysis

	Total (*n* = 714)		0–6 month (*n* = 321)	7–24 months (*n* = 393)
	N	%	%	%
Sex				
Male	393	55.0	53.6	56.2
Female	321	45.0	46.4	43.8
Race				
Chinese	419	58.7	57.3	59.8
Malay	193	27.0	27.1	27.0
Indian	80	11.2	12.2	10.4
Others	22	3.1	3.4	2.8
Parent marital status				
Married	700	98.0	98.1	98.0
Single	14	2.0	1.9	2.0
Father education				
Primary/secondary	147	20.6	17.8	22.9
Post-secondary and above^a^	567	79.4	82.2	77.1
Mother education				
Primary/secondary	173	24.2	22.7	25.4
Post-secondary and above	541	75.8	77.3	74.6
Caregiver				
Parent	524	73.4	80.4	67.7
Relative/others^b^	190	26.6	19.6	32.3
Housing				
Government housing (1–3 rooms)	138	19.3	21.2	17.8
Government housing 4 rooms	329	46.1	42.4	49.1
Government housing ≥5 rooms	216	30.3	33.3	27.7
Condominium/landed property	31	4.3	3.1	5.3
Sleep in hour (Mean, SD)	714	13.9(3.5)	15.6(3.9)	12.4(2.2)

^a^Including post-secondary, diploma and professional qualification, university and post-graduate

^b^Including siblings, relatives, home helpers and others

Average sleep duration of children was reported to be 13.9 (SD = 3.5) hours. For children aged 6 months and below, the average sleep duration was 15.6 (SD = 3.9) hours while 12.4 (SD = 2.2) hours for children aged between 7 to 24 months.

### Screen viewing

Table 2 presents the prevalence of any corresponding screen viewing among total study population, and duration of daily total SV and device-specific SV among those exposed to screen devices. The prevalence of any daily SV was 53.1%. Prevalence of any daily TV (44.3%) and mobile devices (30.1%) viewing was higher than computers (6.6%) and video consoles (0.4%). Due to the low prevalence of video console use, it was not presented in the table.

**Table 2:** Prevalence and duration of total and device-specific screen viewing (SV) among children exposed to screen devices by age

	Count n (%)^a^			Time (Hours/day)^b^		
				Median (IQR)		
	Total	Up to 6 months	7-24 months	Total	Up to 6 months	7-24 months
SV	379 (53.1)	91 (28.3)	288 (73.3)	1.00 (0.50–2.00)	1.00 (0.44–2.00)	1.04 (0.50–2.31)
TV	316 (44.3)	76 (23.7)	240 (61.1)	0.98 (0.43–2.00)	0.80 (0.36–1.86)	1.00 (0.50–2.00)
Computer	47 (6.6)	7 (2.2)	40 (10.2)	0.50 (0.36–1.00)	0.36 (0.17–0.71)	0.57 (0.36–1.00)
Mobile devices	215 (30.1)	42 (13.1)	173 (44.0)	0.50 (0.25–1.00)	0.39 (0.18–1.00)	0.50 (0.29–1.00)

^a^Prevalence of any corresponding screen viewing among total study population; ^b^ Screen viewing duration among children exposed to screen devices

Among children exposed to any screen devices, a median of 1.00 (IQR: 0.50–2.00) hour SV per day was observed. Across various devices, TV viewing time was the highest with a median of 0.98 (IQR: 0.43–2.00) hour per day among TV viewers followed by mobile devices (median = 0.50, IQR: 0.36–1.00) and computer viewing (median = 0.50, IQR: 0.25–1.00) hour per day among children exposed to these devices. Older children (7–24 months) had a much higher prevalence of SV when compared to younger children (up to 6 months): 73.3% vs 28.3%, and this persisted across device-specific SV. SV duration was similar among screen viewers from both groups.

### Association of screen time and sleep duration

Table 3 shows the results of multiple linear regression analyses on the association of total and device- specific SV with sleep duration among the total study population. In univariate analysis (e.g. Model 1), total SV was observed to be negatively associated with sleep duration. The association remained statistically significant, although somewhat attenuated, after controlling for socio-demographic factors in Model 2 (0 h: β = ref.; < 1 h of SV: β = − 1.01 h, 95%CI: -1.63, − 0.40; 1–2 h of SV: β = − 1.42 h, 95%CI: -2.10, − 0.73; > 2 h of SV: β = − 1.73 h, 95%CI: -2.43, − 1.02). This association had a significant dose-response relationship. Similar associations were observed for total SV, TV and mobile devices.

**Table 3:** Associations of total and device-specific screen viewing (hours per day) with sleep duration (hours per day) among all participants

All participants (*n* = 714)	β (95%CI)				*P* value^c^	β (95%CI)	P for trend^d^
SV	0 h (*n* = 335)	< 1 h (*n* = 147)	1–2 h (*n* = 116)	≥ 2 h (*n* = 116)		Per Hour	
Model 1^a^	Ref	−2.03 (− 2.65,-1.41)	− 2.73 (− 3.41,-2.06)	−3.11 (− 3.78,-2.43)	< 0.01	− 0.54 (− 0.68,-0.39)	< 0.01
Model 2^b^	Ref	− 1.01 (− 1.63,-0.40)	− 1.42 (− 2.10,-0.73)	−1.73 (− 2.43,-1.02)	< 0.01	− 0.26 (− 0.40,-0.12)	< 0.01
TV	0 h (*n* = 398)	< 1 h (*n* = 158)	1–2 h (*n* = 77)	≥ 2 h (*n* = 81)		Per Hour	
Model 1^a^	Ref	−1.84 (− 2.44,-1.23)	−2.41 (− 3.21,-1.61)	−2.58 (− 3.37,-1.80)	< 0.01	− 0.69 (− 0.92,-0.46)	< 0.01
Model 2^b^	Ref	−0.78 (− 1.37,-0.20)	−1.27 (− 2.05,-0.48)	−1.32 (− 2.08,-0.56)	< 0.01	− 0.28 (− 0.50,-0.06)	0.01
Mobile devices	0 h (*n* = 499)	< 1 h (*n* = 141)	1–2 h (*n* = 47)	≥ 2 h (*n* = 27)		Per Hour	
Model 1^a^	Ref	−1.94 (− 2.57,-1.32)	−2.43 (− 3.43,-1.44)	−2.19 (− 3.48,-0.90)	< 0.01	−0.69 (− 0.97,-0.41)	< 0.01
Model 2^b^	Ref	−0.85(− 1.45,-0.25)	−0.95 (− 1.90,-0.01)	−1.10 (− 2.32,0.11)	0.01	−0.35 (− 0.61,-0.09)	0.01

^a^Unadjusted model

^b^Multivariable model adjusted for children’s age race, sex, mother’s education, father’s education, marital status, caregiver, housing type

^c^Overall effect of categorical screen viewing variables; ^d^ Trend of association using continuous screen viewing variables

We observed significant interactions for age group and total SV, TV and SV on mobile devices as continuous variables in relation to sleep duration (P for interaction < 0.01, < 0.01, 0.03, respectively). Table 4 provides the results of the analysis stratified by age. Stronger associations between SV and sleep duration were reported among the younger children as compared to older children across different device types, after taking socio-demographic factors into account. Among the younger children, compared with no SV, a total SV time of up to 1 h (β = − 1.59 h, 95%CI: -2.84, − 0.34), 1 to 2 h (β = − 2.07 h, 95%CI: -3.74, − 0.40) and 2 or more hours (β = − 2.88 h, 95%CI: -4.50, − 1.26) per day were significantly associated with shorter sleep duration. Among the older children, a total SV of 1 to 2 h (β = − 0.84 h, 95% CI: -1.45, − 0.23) and 2 or more hours (β = − 0.91 h, 95% CI: -1.54, − 0.28) per day were associated with shorter sleep duration when compared with no SV. A significant dose-response relationship was detected for both age groups (up to 6 months: P for trend < 0.01, 7–24 months: P for trend = 0.03). Similarly, TV viewing of up to 1 h (β = − 1.76 h, 95% CI: -3.05, − 0.48), 1 to 2 h (β = − 2.02 h, 95% CI: -4.01, − 0.04) and 2 or more hours (β = − 2.47 h, 95% CI: -4.29, − 0.65) per day were significantly associated with shorter sleep duration when compared with no SV (P for trend < 0.01) among the younger children. However, among the older children, only TV viewing of 1 to 2 h (β = − 0.68 h, 95% CI: -1.33, − 0.02) was significantly associated with a shorter sleep duration when compared with no SV. With regard to SV on mobile devices among younger children, 1 to 2 h (β = − 3.21 h, 95%CI: -6.15, − 0.27) per day was significantly associated with shorter sleep duration (P for trend = 0.02) when compared with no SV. Among older children, a marginally significant association between SV on mobile devices of up to 1 h (β = − 0.62 h, 95%CI: -1.11, − 0.13, overall *P* = 0.07) per day and sleep duration was observed when compared with no SV.

**Table 4:** Associations of total and device-specific screen viewing (hours per day) with sleep duration (hours per day) stratified by age group

	β (95%CI)				*P* value ^c^	β (95% CI)	P for trend ^d^
Up to 6 months							
SV	0 h (*n* = 230)	< 1 h (*n* = 43)	1–2 h (*n* = 23)	≥ 2 h (*n* = 25)		Per Hour	
Model 1^a^	Ref	−1.75 (− 2.99,-0.51)	−2.26 (− 3.89,-0.63)	−3.26 (− 4.83,-1.69)	< 0.01	− 0.82 (− 1.19,-0.44)	< 0.01
Model 2^b^	Ref	− 1.59 (− 2.84,-0.34)	−2.07 (− 3.74,-0.40)	−2.88 (− 4.50,-1.26)	< 0.01	− 0.73 (− 1.12,-0.34)	< 0.01
TV	0 h (*n* = 245)	< 1 h (*n* = 41)	1–2 h (*n* = 16)	≥ 2 h (*n* = 19)		Per Hour	
Model 1	Ref	−2.01 (−3.28,-0.74)	− 2.05 (− 3.99,-0.11)	−2.74 (− 4.54,-0.95)	< 0.01	− 1.03 (− 1.59,-0.46)	< 0.01
Model 2	Ref	−1.76 (− 3.05,-0.48)	−2.02 (− 4.01,-0.04)	−2.47 (− 4.29,-0.65)	< 0.01	−0.85 (− 1.43,-0.27)	< 0.01
Mobile devices	0 h (*n* = 279)	< 1 h (*n* = 29)	1–2 h (*n* = 7)	≥ 2 h (*n* = 6)		Per Hour	
Model 1	Ref	−1.63 (−3.11,-0.15)	−3.80 (− 6.71,-0.89)	− 2.79 (− 5.93,0.35)	< 0.01	−0.81 (− 1.39,-0.23)	0.01
Model 2	Ref	−1.37 (−2.88,0.14)	−3.21 (− 6.15,-0.27)	− 2.62 (− 5.85,0.61)	0.03	− 0.73 (− 1.33,-0.14)	0.02
7–24 months							
SV	0 h (*n* = 105)	< 1 h (*n* = 104)	1–2 h (*n* = 93)	≥ 2 h (*n* = 91)		Per Hour	
Model 1	Ref	−0.35 (− 0.93,0.23)	−0.88 (− 1.48,-0.28)	− 1.12 (− 1.72,-0.52)	< 0.01	−0.17 (− 0.28,-0.06)	< 0.01
Model 2	Ref	−0.29 (− 0.87,0.29)	−0.84 (− 1.45,-0.23)	−0.91 (− 1.54,-0.28)	0.01	− 0.13 (− 0.24,-0.01)	0.03
TV	0 h (*n* = 153)	< 1 h (*n* = 117)	1–2 h (*n* = 61)	≥ 2 h (*n* = 62)		Per Hour	
Model 1	Ref	−0.16 (− 0.68,0.36)	−0.77 (− 1.41,-0.13)	−0.86 (− 1.50,-0.23)	0.01	− 0.17 (− 0.34,0.01)	0.06
Model 2	Ref	− 0.08 (− 0.60,0.43)	−0.68 (− 1.33,-0.02)	−0.61 (− 1.26,0.05)	0.1	−0.09 (− 0.27,-0.08)	0.29
Mobile devices	0 h (*n* = 220)	< 1 h (*n* = 112)	1–2 h (*n* = 40)	≥ 2 h (*n* = 21)		Per Hour	
Model 1	Ref	−0.62 (− 1.12,-0.13)	− 0.68 (− 1.41,-0.05)	−0.66 (− 1.62,0.31)	0.04	−0.23 (− 0.45,-0.02)	0.04
Model 2	Ref	−0.62 (− 1.11,-0.13)	−0.54 (− 1.28,0.19)	−0.45 (− 1.43,0.53)	0.07	− 0.17 (− 0.40,-0.05)	0.12

^a^Unadjusted model

^b^Multivariable model adjusted for children’s age race, sex, maternal education, father’s education, mother’s education, caregiver, marital status, housing type

^c^Overall effect of categorical screen viewing variables

^d^Trend of association using screen viewing variables

## Discussion

To our knowledge, our study is the first to investigate the association between device-specific SV and sleep in very young children from a multiethnic Asian population. We found that longer total and device-specific SV was associated with shorter sleep duration among children aged 2 years and below. Associations were stronger for children aged 6 months and below when compared to children aged 7 to 24 months**.**

In our study, TV was still the most commonly used screen device contributing the highest viewing time among these very young children in Singapore. However, more than half of children also used mobile devices actively. On average, this study reported an adequate daily sleep duration among the very young children. Compared with other high-income countries in Asia, sleep duration reported in this study is similar to that among Taiwanese young children (13.7 h/day) [57], and more than that among infants and toddlers in Japan (11.6 h/day) [58] and South Korea (11.9 h/day) [59]. However, unlike the high compliance reported among Australian (88.7%) [60] and Canadian (83.9%) toddlers [61], almost half of our study sample failed to meet the sleep recommendations [62, 63].

There was a strong inverse association between SV and sleep in children aged up to 2 years, to the extent that children who had more than 1 h of total SV per day slept 1.5 h less than those without any SV. Our findings suggested that associations were even stronger in younger children as compared to older ones. We hypothesize that screen devices may affect sleep through several pathways. Firstly, the use of screen devices may directly lead to a reduction of sleep duration by delaying in naptime and bedtime [64]. Secondly, viewed content may be psychologically stimulating thereby leading to delayed sleep or interruptions of nighttime sleep [65]. Thirdly, exposure to bright light of screen devices before sleep may interrupt the sleep-wake cycle by suppressing the release of the hormone melatonin [66]. Consequently, the onset of nighttime sleepiness may be postponed due to the increasing alertness caused by bright light [67]. These findings were consistent with existing evidence from previous studies where the exposure to and use of screen media was negatively associated with nighttime sleep duration among school-aged children [37, 42, 68] and adolescents [69, 70]. Our study found that SV from two common types of screen device use (TV and mobile devices) had negative associations with sleep duration, expanding on previous studies investigating the relationship of single SV device and napping and nighttime sleep among young children [64, 71]. In the present study, we observed 0.28 h’ shorter sleep duration per 1-h increase in TV viewing among all children, with a considerably stronger association among younger children. The magnitude of association in our study is similar to that reported in the recent longitudinal study of TV viewing and sleep duration among 1864 American children, each 1 h longer TV viewing per day from infancy to mid-childhood was associated with fewer hours of total sleep duration (including napping and nighttime sleep) during the same period (0.12 h per day, 95%CI: 0.07, 0.17) [46]. Consistent with our finding that SV on mobile devices was significantly associated with shorter sleep duration in young children, a previous systematic review reported a strong and consistent positive relationship between bedtime mobile device use and inadequate nighttime sleep (OR: 2.17; 95% CI: 1.42, 3.32) among school-age children [72]. In our study, increasing SV on mobile devices was associated with similar reductions in sleep duration as compared to TV viewing. Traditional SV devices such as TVs have been used by parents as a way for their children to rest, as a babysitter [73] and a behavior management tool [74]. In addition to these functions, mobile screen devices, including phones and tablets are portable with broader capabilities resulting in a different level and type of exposure than TVs, as these mobile screen devices enable continuous stimulation for children by allowing real-time interaction [72].

Our study makes a unique contribution to the existing literature. To our knowledge, existing studies on SV and sleep have not assessed multiple screen devices among very young children. However, the variety of SV exposures reflects modern SV patterns. Our study also has a very high participation rate and the characteristic of the children in our study was broadly comparable to the children population in Singapore [49]. However, there are also some methodological limitations to be acknowledged. Firstly, for logistical and administrative reasons, participants were recruited from 2 out of 18 polyclinics in Singapore. Although there is no information suggesting that there may be a difference between polyclinics, such differences may still exist which would decrease generalizability of our findings. Secondly, our study adopted a subjective measure, a proxy questionnaire, to determine SV and sleep duration. All data was collected by interviewers rather than self-administered and respondents did not have difficulty understanding the questions*.* Although our questionnaire was developed based on existing literature and piloted extensively, it was not validated in the target population. This may introduce measurement error which could attenuate the associations towards the null. Interviewer-administered questionnaires can be affected by various response biases, such as social desirability and recall bias. Although children’s demographics (i.e. age, sex, race) and family characteristics (i.e. caregivers, marital status of parents, housing type and paternal and maternal educational levels) were adjusted in the analysis, residual confounding might still exist due to unmeasured factors such as children’s anthropometrics and parenting style. Thirdly, since this was a cross-sectional study, it was unable to fully conclude the direction of association between SV time and sleep duration. Fourthly, a one-item question was used to determine sleep duration, which might be suboptimal for very young children who tend to take several naps throughout the day. Younger children have been shown to have more wakeups and shorter sleeping cycle when compared with older ones [75], however, the one-item question was not able to differentiate the different sleeping cycles. While this item was derived from a questionnaire that has previously been validated among an infant population and widely used in other studies among infants, toddlers and children [52–54], we acknowledge that objective means of measuring sleep duration, for instance via actigraphy, may be advisable in future studies. In addition, the one-item question did not capture any information of sleep quality, which is another valuable measure of sleep [76]. Finally, young children may benefit from quality programmes or interactive media [77] while some content, such as media violence, can elevate psychological and physiological arousal, making it more difficult for children to fall asleep and resulting in impaired sleep quality of sleep [78]. We did not include more information of the content of media use in this study, which may be important to better explain the mechanisms by which SV impacts sleep patterns.

It is important that caregivers, teachers and healthcare professionals are educated about the potential detrimental influence of SV on sleep which may in turn affect children’s daytime function, heath behavior and lead to health risks in the long run. Based on literature and our findings, we encourage policy-led guidance to communities on the promotion of sleep hygiene. In addition, a checklist for monitoring sleeping behavior of children during routine clinic visits is important in order to explore screen device use as a possible risk factor. Given the evolving technology landscape, parents may believe in the educational and entertainment value of media devices and replace traditional paper books with mobile devices [73]. It is likely that the access and use of media screen devices will increase further among very young children. Our findings support the need for interventions to reduce the access and usage of mobile screen devices, especially among very young children.

Our study provides relevant new evidence for an increasingly important public health issue. The phenomenon of very young children using multiple screen devices is nowadays almost common practice, which could be motivated by parents’ beliefs in the educational values of using screen devices. However, more research is needed to address those beliefs. TVs are not recommended to be placed in a child’s bedroom [79]. Given that mobile devices may exert similar influences on sleep, further research should assess the presence of other screen devices in a child’s bedroom and its association with sleep and other health related outcomes among very young children.

## Conclusions

Our study suggests strong adverse associations between SV time across different screen devices with sleep duration among Singaporean children aged 2 years and below. These associations were particularly strong among children below 6 months of age. Collaborative approaches from caregivers, teachers, and healthcare professionals are needed to reduce screen device access and use among very young children in order to minimize the negative impact on child sleep and potentially child health. Nevertheless, further research is warranted to corroborate our observations and to develop effective intervention strategies to reduce SV among very young children.

## Additional file

Additional file 1: Items on device-specific SV from questionnaire. A supplementary table containing items regarding device-specific SV at home. (DOCX 12 kb)

## Abbreviations

AAP: American Academy of Pediatrics
DSRB: Domain Specific Review Board
SV: Screen viewing
TV: Television
US: United States
