# Supplementary material for: Screen viewing behavior and sleep duration among children aged 2 and below
Source: BMC Public Health. 2019 Jan 14;19:59. doi: 10.1186/s12889-018-6385-6 (PMC6332844; doi:10.1186/s12889-018-6385-6)
Supplement: Supplementary file 1 — Items on device-specific SV from questionnaire. A supplementary table containing items regarding device-specific SV at home. (DOCX 12 kb) [file 12889_2018_6385_MOESM1_ESM.docx]

| **Additional file 1 Items on device-specific SV from questionnaire** | | |
| --- | --- | --- |
| Please estimate the number of hours your child is directly exposed to the following electronic items **at home**. If you do not own any of the following items, please write the number "0” | | |
|  | Single weekday | Single weekend |
| Televisions/DVD/VCR | ___h:___min | ___h:___min |
| Computers (including desktops, laptops and ultra-books) | ___h:___min | ___h:___min |
| Video game consoles | ___h:___min | ___h:___min |
| Mobile phones, tablets, handheld video game devices e.g. PSP, Nintendo DS | ___h:___min | ___h:___min |
